# 

**Published:** 1868-11-01

**Authors:** 


### *CONTENTS:*

ORIGINAL PAPERS:

Leucocytes. Can they be developed spontaneously in Blastemata? Origin of Leucocytes found in the midst of Blastemata primarily amorphous, isolated in permeable receptacles. Translated by Walter Hay, M.D., Associate Editor, Chicago.... 679

Modern Treatment of Acute Internal Inflammations.... 686

PHILADELPHIA CORRESPONDENCE.... 705

EDITORIAL:

Apologetic.... 708

Gettysburg Kaalysine Water.... 709

